# NO and NO_2_ Sensing Properties of WO_3_ and Co_3_O_4_ Based Gas Sensors

**DOI:** 10.3390/s130912467

**Published:** 2013-09-17

**Authors:** Takafumi Akamatsu, Toshio Itoh, Noriya Izu, Woosuck Shin

**Affiliations:** National Institute of Advanced Industrial Science and Technology (AIST), Advanced Manufacturing Research Institute, 2266-98 Anagahora, Shimo-Shidami, Moriyama-ku, Nagoya 463-8560, Japan; E-Mails: itoh-toshio@aist.go.jp (T.I.); n-izu@aist.go.jp (N.I.); w.shin@aist.go.jp (W.S.)

**Keywords:** gas sensor, metal oxide semiconductor, diffuse reflectance infrared Fourier transform spectroscopy, NO_2_, NO

## Abstract

Semiconductor-based gas sensors that use n-type WO_3_ or p-type Co_3_O_4_ powder were fabricated and their gas sensing properties toward NO_2_ or NO (0.5–5 ppm in air) were investigated at 100 °C or 200 °C. The resistance of the WO_3_-based sensor increased on exposure to NO_2_ and NO. On the other hand, the resistance of the Co_3_O_4_-based sensor varied depending on the operating temperature and the gas species. The chemical states of the surface of WO_3_ or those of the Co_3_O_4_ powder on exposure to 1 ppm NO_2_ and NO were investigated by diffuse reflectance infrared Fourier transform (DRIFT) spectroscopy. No clear differences between the chemical states of the metal oxide surface exposed to NO_2_ or NO could be detected from the DRIFT spectra.

## 1. Introduction

Since environmentally hazardous gases include toxic and greenhouse effect gases, the threshold limit value, which is defined as the maximum concentration of a chemical allowable for repeated exposure without producing adverse health effects, is regulated by the American Conference of Governmental Industrial Hygienists [1]. Effective and inexpensive systems for the detection and quantification of environmentally hazardous gases are required. Standard air pollution measurements are still based on time-consuming and expensive analytical techniques such as optical spectroscopy and gas chromatography [2,3]. Gas sensors have been considered as promising candidates for measurement of environmental pollution levels because of their low cost, high sensitivity, fast response, and direct electronic interface.

Environmentally hazardous gases can be classified into oxidizing gases (such as NO_2_, CO_2_, and Cl_2_) and reducing gases (such as NO, H_2_S, CO, and C_2_H_5_OH). When an oxidizing gas is steamed on an n-type semiconductor surface, the concentration of electrons on the surface decreases and the resistance of the n-type semiconductor increases. In the case of a p-type semiconductor, the concentration of electrons on the p-type semiconductor surface decreases and the resistance of the p-type semiconductor decreases because the extracted electrons result in the generation holes in the valence band. When the reducing gas is streamed on a metal oxide semiconductor, the gas reacts with the oxygen ions on the semiconductor surface, releasing electrons back to the conduction band. Therefore, when the concentration of electrons on the semiconductor surface increases, the resistance of the n-type semiconductor decreases and that of the p-type semiconductor increases because the generated electrons recombine with holes [4].

Many kinds of NO*_x_* (NO and NO_2_) gas sensors including metal oxide semiconductors [5–7] and solid electrolytes [8,9] have been investigated. Among metal oxide semiconductors, n-type semiconductors, specifically those based on WO_3_, which are highly sensitive, are promising candidates that can be used for the detection of NO*_x_* gas [10–12]. Despite the large number of reports on the use of metal oxide semiconductors for the detection of NO*_x_*, only a few make a clear distinction between the response toward NO and NO_2_. Because NO is easily oxidized to NO_2_ in air, the detection of NO_2_ gas is carried out via the oxidation of NO by an oxidizing agent such as alumina supported potassium permanganate or by oxygen in air over a catalyst such as Pt [13,14]. To develop a high-performance NO gas sensor, it is essential to understand the means of optimizing the semiconductor that constitutes the sensor.

In the present work, the gas sensing properties of the sensor element that uses both n-type WO_3_ and p-type Co_3_O_4_ toward the NO_2_ and NO were examined. The oxidation of NO was a thermally activated reaction in air atmosphere so that the *in situ* observation of the chemical state of NO_2_ and NO on the surface of the sensing material at the temperature of operation may provide important information on the gas detection mechanism. A diffuse reflectance infrared Fourier transform (DRIFT) spectroscopy is an excellent analysis to obtain the chemical state on the surface of the sensing material and has been performed to elucidate the gas detection mechanism of the gas sensors [15,16]. The chemical states of NO_2_ and NO on the surface of the semiconductor oxide were investigated by the DRIFT spectroscopy at a specific working temperature could be changed to indicate the two temperature used in this project.

## 2. Experimental

WO_3_ powder (99.5%, Wako Pure Chemical, Osaka, Japan) and Co_3_O_4_ powder (average particle size: 20∼30 nm, 99.8%, Sigma-Aldrich, St. Louis, MO, USA) were mixed with an organic dispersant, consisting of a mixture of ethyl cellulose and terpineol, to obtain a paste. The weight ratio of the mixture of ethyl cellulose and terpineol was 1:9. The weight ratio of the powder and the organic dispersant was 1:16. The paste was dispensed on a 5 × 9.5 mm^2^ surface-oxidized Si substrate, which consisted of a 2.5 × 4 mm^2^ platinum comb-type electrode with a gap and line width of 10 μm each. The substrate was annealed at 500 °C for 3 h under air to obtain the sensor elements consisting of n-type WO_3_ and p-type Co_3_O_4_.

The cross-sectional SEM image of the sensor element of WO_3_ or Co_3_O_4_ was observed using field-emission scanning electron microscopy (FE-SEM; JSM-6335FM, JEOL, Tokyo, Japan). For the cross-sectional observations, the sensor element was broken at the center of the WO_3_ of Co_3_O_4_ film to form the sample. The thickness of the WO_3_ or Co_3_O_4_ film was estimated from the cross-sectional FE-SEM image.

**Figure 1.**
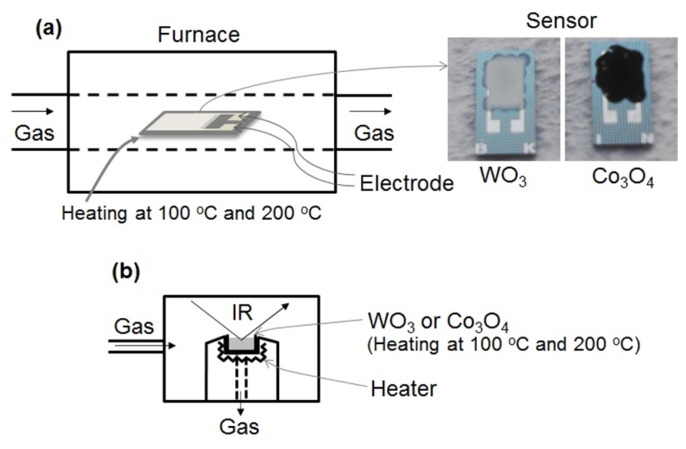
Schematic drawings of the experimental apparatus used (**a**) For measuring the gas sensing properties and (**b**) For carrying out DRIFT spectroscopy.

Figure 1 shows the schematic drawings of the experimental apparatus used for measuring the gas sensing properties and to carry out DRIFT spectroscopy. The gas sensing properties of the sensor element was measured using a flow-type gas sensing measurement apparatus as shown in Figure 1a. The element was placed in a tubular sample chamber heated to 100 °C or 200 °C in an electrical tube furnace. Air was introduced into the chamber for 15 min, and then, a gas mixture of NO_2_ or NO in air was injected for 15 min. Then the flow of gas mixture was halted and replaced by air injected at a flow rate of 200 mL/min. The concentration of NO_2_ or NO was controlled to take the values of 0, 0.5, 1, and 5 ppm in air. The electrical resistance of the element in various gaseous atmospheres was measured by a multimeter (K2700, Keithley, Cleveland, OH, USA). The electrical resistances of the sensor in air and under the gas mixture are denoted as *R_a_* and *R_g_*, respectively. The value of *R_g_* of the element was measured after 15 min of exposure to NO_2_ or NO gas. When *R_g_* is higher than *R_a_*, the sensor response is defined as *S* = *R_g_*/*R_a_*. However, when *R_g_* is lower than *R_a_*, the response is defined as *S* = *R_a_*/*R_g_*.

The DRIFT spectrum of the WO_3_ and Co_3_O_4_ powder were recorded by a spectrometer (Nexus 470 FTIR, Nicolet, Waltham, MA, USA) equipped with liquid nitrogen cooled MCT detector, as shown in Figure 1b. After loading the powder into the DRIFT sample cell, the powder was purged under air flow and heated at 350 °C to eliminate impurities on the powder surface. Then, the powder was cooled at 100 °C or 200 °C to obtain the interferogram, which was used as the background reference for the DRIFT spectrum. Following this, 1 ppm NO or NO_2_ in air was introduced into the powder and the DRIFT spectrum was obtained. Then, the powder was purged to remove NO or NO_2_ by air flow and the DRIFT spectrum was once again obtained. The spectra were recorded at a spectral resolution of 1 cm^−1^ with 256 scans.

## 3. Results and Discussion

### 3.1. Gas Sensing Properties of WO_3_

**Figure 2.**
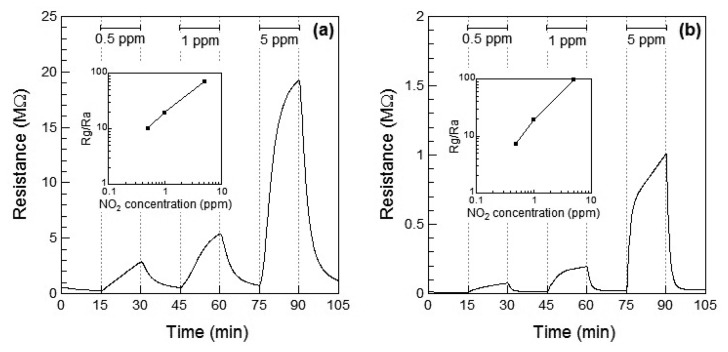
Response of the sensor element using WO_3_ powder during exposure to NO_2_ (0, 0.5, 1, and 5 ppm) in air at (**a**) 100 °C and (**b**) 200 °C. The inset shows the relationship between the sensor response and NO_2_ concentration.

Figure 2a,b show the response of the WO_3_ sensor element to NO_2_ at 100 °C and 200 °C, respectively. When NO_2_ gas was introduced, the resistance of the WO_3_ sensor element increased with increase in the concentration of NO_2_. This is the typical response of an n-type oxide toward an oxidizing gas, leading to *R_g_* > *R_a_*. At 200 °C, *R_a_* of WO_3_ decreased with temperature, and the sensor responses were higher than those at 100 °C. At 200 °C, the response of the WO_3_ sensor element increased to *S* = 19.2 at 1 ppm of NO_2_ and the response was adequately linear. The response and recovery times of the resistance of the WO_3_ sensor element reduced with increasing the operating temperature, but the resistance did not reach the saturation even after 15 min for NO_2_ exposure. The response time of the WO_3_ sensor element was not so fast in comparison with the other sensors [5–7]. In order to reduce the response and recovery times, the optimum operating temperature is required.

**Figure 3.**
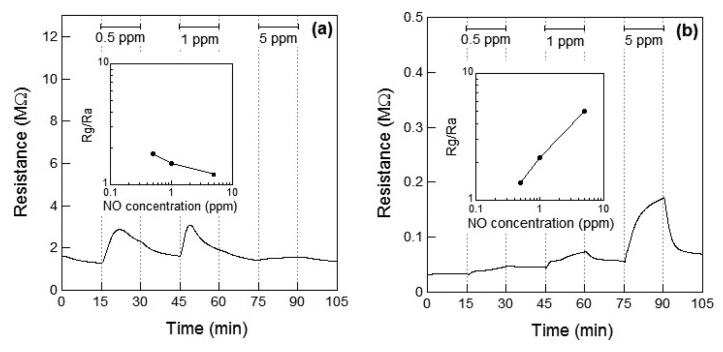
Response of the sensor element using WO_3_ powder during exposure to NO (0, 0.5, 1, and 5 ppm) in air at (**a**) 100 °C and (**b**) 200 °C. The inset shows the relationship between the sensor response and NO concentration.

Figure 3a,b show the response of the WO_3_ sensor element to NO at 100 °C and 200 °C, respectively. When NO gas was introduced at 100 °C, the resistance of the sensor element immediately increased and then subsequently decreased within 10 min. No clear relationship between the gas concentration and sensor response could be observed. Therefore, the resistance of the sensor to 5 ppm of NO was smaller than that to 0.5 and 1 ppm NO. The unexpected changes of the sensor resistance of WO_3_ are supposed to be under the influence of the low operating temperature of 100 °C, which is not high enough for desorption of the reaction product on the WO_3_. However, when the WO_3_ sensor was exposed to NO gas at 200 °C, the resistance of the element increased with increase in the concentration of NO, similar to the response shown to NO_2_, as seen in Figure 2b. At 200 °C, the response of the WO_3_ sensor element to 1 ppm NO was *S* = 2.2, and the response was adequately linear.

The resistance of the WO_3_ sensor element increased by exposure to NO_2_ and NO at 200 °C. Although the resistance of the sensor based on the n-type WO_3_ is considered to be decreased by exposure to reductive NO, the resistance increased, as shown in Figure 3a,b. Since NO with an unpaired electron is unstable state, NO easily reacts with oxygen in air to become a stable NO_2_. The reaction of NO to NO_2_ proceeds with temperature [13]. It has previously been reported that NO could be partially oxidized to NO_2_ and leading to adsorption of NO_2_ on WO_3_ (or Pt-doped WO_3_) surface, which has been confirmed by temperature programmed desorption (TPD) analysis [17]. Therefore, we assumed that the resistance of the sensor on exposure to NO increased and the responses of the sensor toward NO exposure were smaller than those toward NO_2_ exposure at 100 °C and 200 °C because of the partial oxidation of NO.

### 3.2. Gas Sensing Properties of Co_3_O_4_

**Figure 4.**
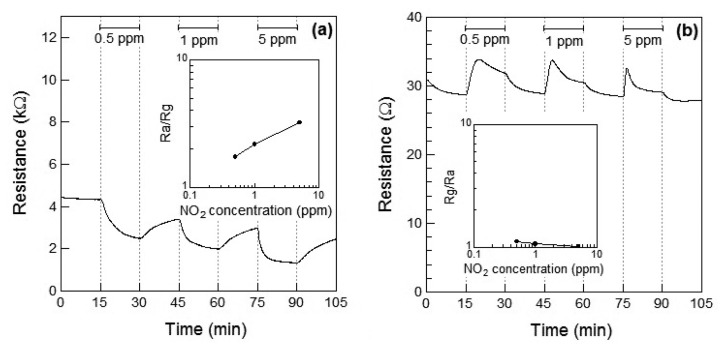
Response of the sensor element using Co_3_O_4_ powder during exposure to NO_2_ (0, 0.5, 1, and 5 ppm) in air at (**a**) 100 °C and (**b**) 200 °C. The inset shows the relationship between the sensor response and NO_2_ concentration.

Figure 4a,b show the response of the Co_3_O_4_ sensor element toward NO_2_ exposure at 100 °C and 200 °C, respectively. When NO_2_ gas was introduced at 100 °C, the resistance of the sensor element immediately decreased. This may be attributed to the adsorption of NO_2_ onto the surface of the p-type semiconductor Co_3_O_4_ and to the role of NO_2_ as an oxidizing gas at 100 °C. A linear relationship between the gas concentration and the sensor response was observed. The response of the Co_3_O_4_ sensor element to 1 ppm of NO_2_ at 100 °C was *S* = 2.2. The sensor resistance of NO_2_-exposed Co_3_O_4_ did not reached to *R_a_* even after 15 min for air exposure; the recovery time of Co_3_O_4_ sensor element was not so fast. In our preliminary experiment, the sensor resistance of NO_2_-exposed Co_3_O_4_ reached the saturation within 60 min for air exposure. Therefore, the sensor resistance of NO_2_-exposed Co_3_O_4_ in this work is also expected to reach the saturation within 60 min for air exposure. When the sensor was exposed to NO_2_ at 200 °C, the resistance of the sensor element immediately increased and then gradually decreased on further exposure to NO_2_, which seems to indicate the role of NO_2_ as a reducing gas at 200 °C. The decrease in the resistance of the Co_3_O_4_ sensor increased with the concentration of the NO_2_ gas. As a result, the resistance of the Co_3_O_4_ element after 15 min of exposure to NO_2_ decreased with the concentration of NO_2_ gas. In our preliminary experiment, the sensor resistance of the Co_3_O_4_ reached the saturation after 30 min for NO_2_ exposure and did not reached to *R_a_* even after 60 min for NO_2_ exposure. Therefore, the sensor resistance of the Co_3_O_4_ in this work is also expected to reach the saturation within 30 min for NO_2_ exposure. No clear linear relationship between the gas concentration and sensor response could be observed.

**Figure 5.**
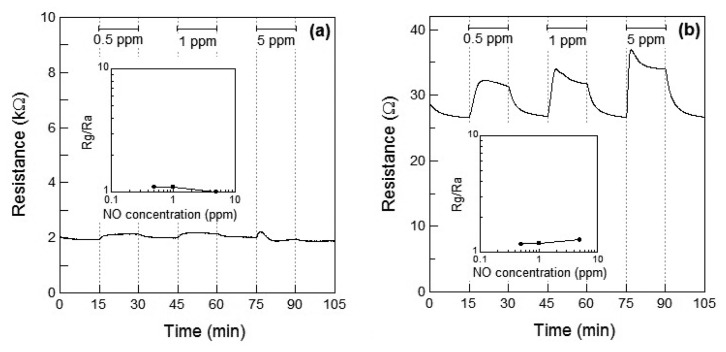
Response of the sensor element using Co_3_O_4_ powder during exposure to NO (0, 0.5, 1, and 5 ppm) in air at (**a**) 100 °C and (**b**) 200 °C. The inset shows the relationship between the sensor response and NO concentration.

Figure 5a,b show the response of the Co_3_O_4_ sensor element at 100 °C and 200 °C, respectively, toward NO. When 5 ppm of NO gas was introduced at 100 °C, the resistance of the Co_3_O_4_ sensor slightly increased. However, the resistance decreased within 3 min of exposure and a linear relationship between the NO gas concentration and the resistance of the Co_3_O_4_ sensor element was absent. However at 200 °C, the resistance increased with the concentration of NO gas and the relationship between the gas concentration and sensor response was linear, as shown in Figure 5b. The response of the Co_3_O_4_ sensor element to 1 ppm NO at 200 °C was *S* = 1.2.

Figure 4a shows the *R_g_* decrease exhibited by the p-type Co_3_O_4_ on exposure to NO_2_ at 100 °C, however, the response of resistance *R_g_* of Co_3_O_4_ reversed and *R_g_* increased on exposure to NO_2_ at 200 °C. This contradictory result indicated a role of NO_2_ as an oxidizing gas at 100 °C and as a reducing gas at 200 °C. However, it rermains unclear how an oxidizing gas such as NO_2_ could become a reducing gas at a different temperature. As shown in Figure 5a,b, NO is expected to be adsorbed on the surface of Co_3_O_4_ and act as a reducing gas, resulting in an increase in the sensor resistance. The sensor resistance of Co_3_O_4_ on exposure to 5 ppm of NO at 100 °C increased within 3 min of exposure and then became slightly smaller than *R_a_*. The unexpected changes of the sensor resistance of Co_3_O_4_ are supposed to be due to insufficient desorption of reaction product on the oxide surface, similar to the case of the WO_3_ sensor. The reaction of Co_3_O_4_ on exposure to 5 ppm of NO at 100 °C is assumed as follows: NO reacts with adsorbed oxygen on Co_3_O_4_ surface and the sensor resistance increases; the reaction of NO and adsorbed oxygen generates NO_2_. Although the generated NO_2_ is normally desorbed from Co_3_O_4_ surface, the generated NO_2_ is expected to be readsorbed into Co_3_O_4_ surface at 100 °C; the sensor resistance of Co_3_O_4_ gradually decreases.

**Figure 6.**
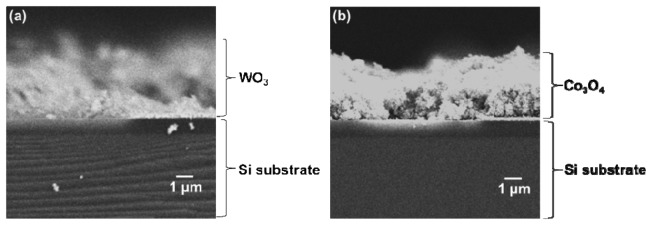
Cross-sectional SEM images of the sensor element of (**a**) WO_3_ and (**b**) Co_3_O_4_.

Figure 6a,b show cross-sectional SEM images of the sensor element of WO_3_ and Co_3_O_4_. The thicknesses of WO_3_ and Co_3_O_4_ films were estimated to be 3.6 and 3.3 μm, respectively. We reported the relationship between the gas response toward nonanal gas and Pt-, Pd-, Au-loaded SnO_2_ film [18]. In that report, the sensor response toward nonanal gas was influenced by the thickness of Pt-, Pd-, Au-loaded SnO_2_ film and also the operating temperature. Although the sensor responses of WO_3_ and Co_3_O_4_ are not sufficiently large at this stage, we expect that they can be improved after optimizing the thicknesses of WO_3_ and Co_3_O_4_ films and the operating temperature.

### 3.3. DRIFT Spectra of WO_3_ Powder

**Figure 7.**
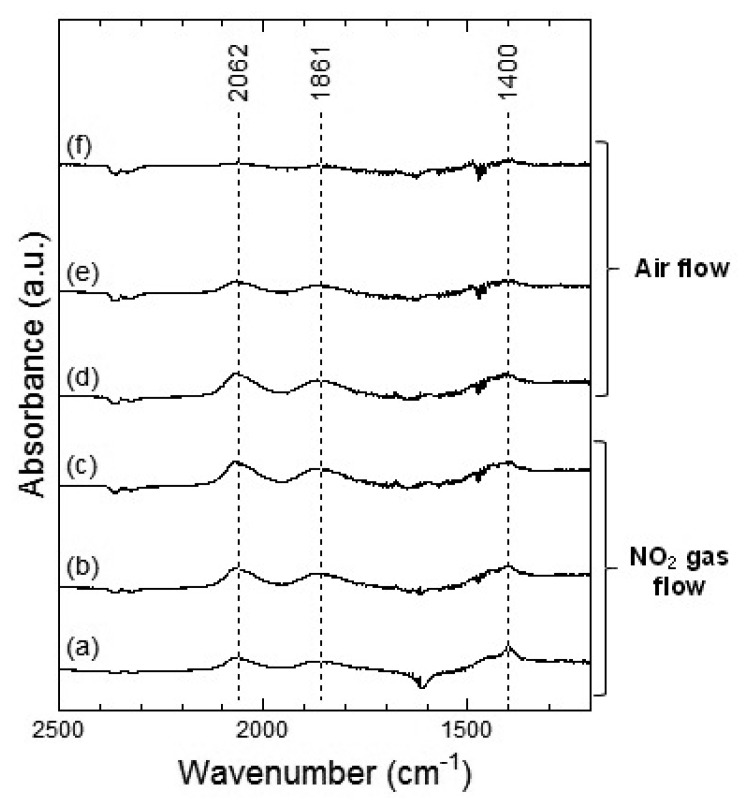
DRIFT spectra of WO_3_ powder at 100 °C. The powders were exposed to 1 ppm of NO_2_ in air for (**a**) 2 min, (**b**) 20 min, and (**c**) 50 min. After NO_2_ exposure, the powders were purged by air for (**d**) 1 min, (**e**) 30 min, and (**f**) 60 min.

**Figure 8.**
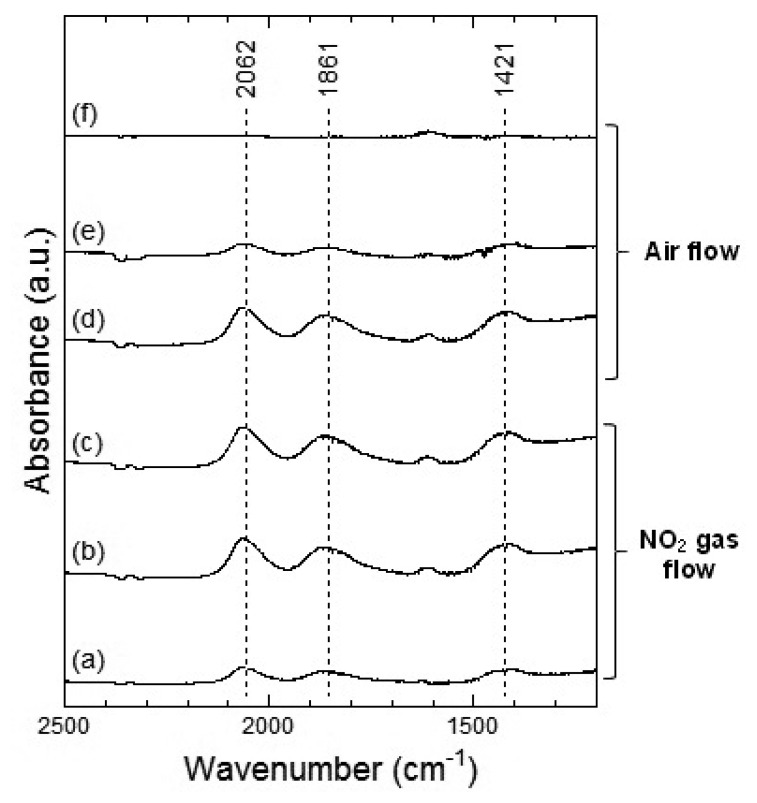
DRIFT spectra of WO_3_ powder at 200 °C. The powders were exposed to 1 ppm of NO_2_ in air for (**a**) 2 min, (**b**) 20 min, and (**c**) 50 min. After NO_2_ exposure, the powders were purged by air for (**d**) 1 min, (**e**) 30 min, and (**f**) 60 min.

Figures 7 and 8 show the DRIFT spectra of WO_3_ powder on exposure to 1 ppm NO_2_ exposure at 100 °C and 200 °C, respectively. As shown in Figure 7, the intensity of three peaks at around 2,062, 1,861 and 1,400 cm^−1^ increased with the time of exposure of NO_2_ and decreased with purging with air. According to reports, the peaks around 2,062 and 1,861 cm^−1^ can be assigned to various overtones and combination modes of the W-O bond in the lattice of the oxide [16,19]. The several small peaks in the 1,400–1,700 cm^−1^ region seemed to be the result of multiple overlapping bands and could not be determined. The peak around 1,400 cm^−1^ could be assigned to nitrate species [20,21].

At 200 °C, the peaks around 2,062, 1,861 and 1,421 cm^−1^ were also observed in WO_3_ on NO_2_ exposure. The peaks at about 2,062 and 1,861 cm^−1^ formed upon interaction with NO_2_ at 200 °C were similar to those at 100 °C and the peaks could be assigned to the various overtones and combination modes of the bond between oxygen and tungsten in the lattice of the oxide. The peak at around 1,421 cm^−1^ could be assigned to nitrate species. This peak at 200 °C seemed much stronger than that at 100 °C and hence, the amount of the nitrate species on WO_3_ surface at 200 °C was larger than that at 100 °C. When air was introduced to the WO_3_ powder sensor after exposure to NO_2_, the intensity of the peaks at 100 °C and 200 °C decreased with the time of air flow, which indicated the desorption of NO_2_ from the surface of WO_3_.

**Figure 9.**
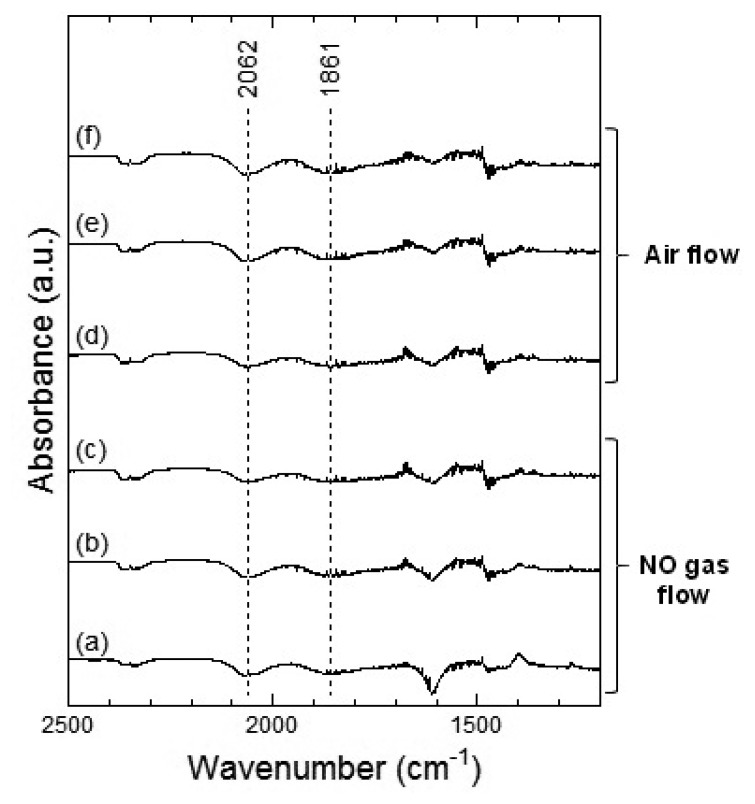
DRIFT spectra of WO_3_ powder at 100 °C. The powders were exposed to 1 ppm of NO in air for (**a**) 2 min, (**b**) 20 min, and (**c**) 50 min. After NO exposure, the powders were purged by air for (**d**) 1 min, (**e**) 30 min, and (**f**) 60 min.

**Figure 10.**
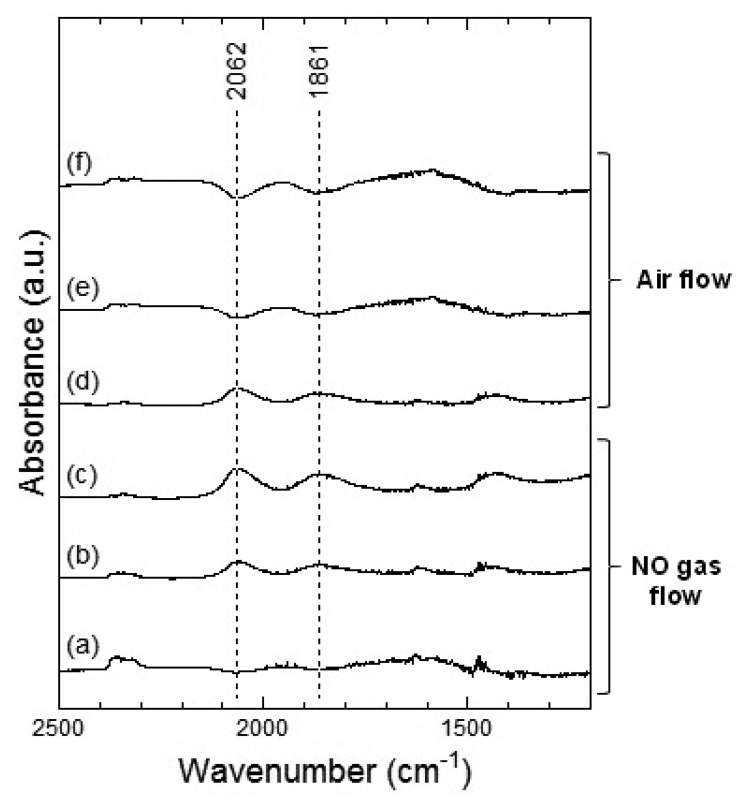
DRIFT spectra of WO_3_ powder at 200 °C. The powders were exposed to 1 ppm of NO in air for (**a**) 2 min, (**b**) 20 min, and (**c**) 50 min. After NO exposure, the powders were purged by air for (**d**) 1 min, (**e**) 30 min, and (**f**) 60 min.

Figures 9 and 10 show the DRIFT spectra of WO_3_ powder on exposure to 1 ppm of NO at 100 °C and 200 °C, respectively. In Figure 9, negative peaks at around 2,062 and 1,861 cm^−1^ were seen upon the introduction of NO gas. WO_3_ powder is supposed to be unreactive for NO at 100 °C because there was no difference between the spectra obtained on exposure to NO (Figure 9a–c) and on exposure to air (Figure 9d–f). However, as shown in Figure 10, the intensity of the peaks at around 2,062 and 1,861 cm^−1^ increased with the time of exposure to NO and decreased on introduction of air. The negative peaks of NO observed in Figure 9, Figure 10e,f may be attributed to possible glitches with the background subtraction. When NO reacts with adsorbed oxygen on the WO_3_ surface, NO_2_ formed and adsorbed on the surface of WO_3_, resulting in the increase in the intensity of the peaks shown in Figure 7 or Figure 8. Subsequently, NO_2_ desorbed from the surface of WO_3_ by air flow, and the intensity of the peaks reduced again.

In the DRIFT spectra of WO_3_ on exposure to NO_2_ and NO, peaks corresponding to NO vibration and NO_2_ asymmetric vibration bands could not be observed. Previous reports have shown the presence of peaks corresponding to CO vibration and CO_2_ asymmetric vibration bands in the DRIFT spectra of SnO_2_ exposed to CO, while peaks of W-O alone were observed in the DRIFT spectra of WO_3_ exposed to CO [16,22]. We assumed that WO_3_ may have insignificant interaction with NO_2_ or NO gas in comparison with other metal oxide semiconductors. The positions of the peaks observed in the DRIFT spectra of WO_3_ exposed to NO_2_ were similar to those observed in the DRIFT spectra of WO_3_ exposed to NO. Further, as shown in Figure 7, the peaks in the DRIFT spectra were small and there were hardly any differences between the DRIFT spectra. Therefore, the response of the sensor to NO exposure was more unstable than that to NO_2_ exposure at 100 °C.

### 3.4. DRIFT Spectra of Co_3_O_4_ Powder

**Figure 11.**
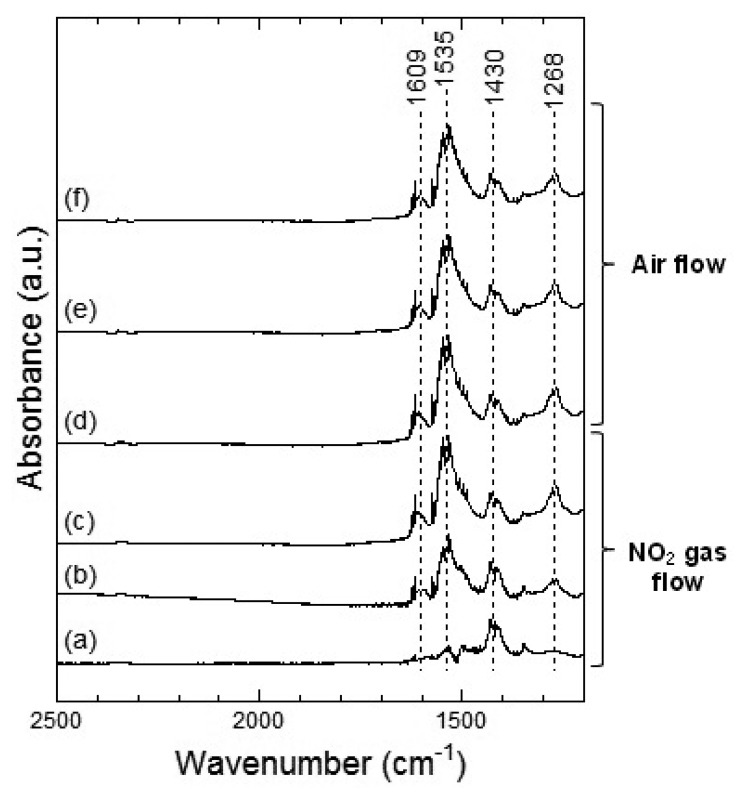
DRIFT spectra of Co_3_O_4_ powder at 100 °C. The powders were exposed to 1 ppm of NO_2_ in air for (**a**) 2 min, (**b**) 20 min, and (**c**) 50 min. After NO_2_ exposure, the powders were purged by air for (**d**) 1 min, (**e**) 30 min, and (**f**) 60 min.

**Figure 12.**
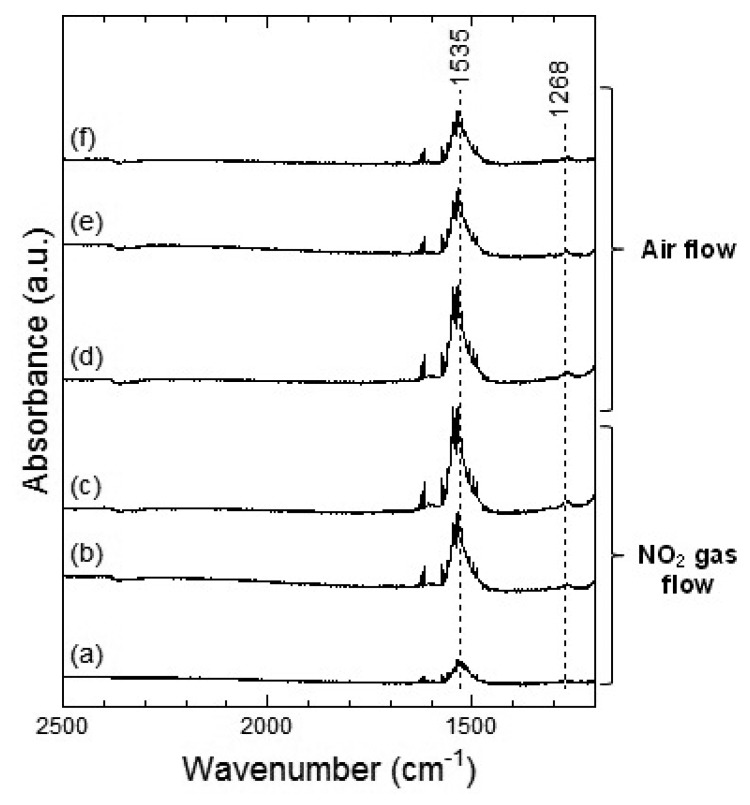
DRIFT spectra of Co_3_O_4_ powder at 200 °C. The powders were exposed to 1 ppm of NO_2_ in air for (**a**) 2 min, (**b**) 20 min, and (**c**) 50 min. After NO_2_ exposure, the powders were purged by air for (**d**) 1 min, (**e**) 30 min, and (**f**) 60 min.

Figures 11 and 12 show the DRIFT spectra of Co_3_O_4_ powder exposed to 1 ppm NO_2_ at 100 °C and 200 °C, respectively. In Figure 11, four peaks around 1,609, 1,535, 1,430 and 1,268 cm^−1^ were observed which are reported to correspond to the NO vibration band of the bridging bidentate nitrate, the NO vibration band of the chelating bidentate nitrate, the NO_2_ asymmetric vibration band of the monodentate nitrate, and the NO_2_ asymmetric vibration of the bridging bidentate nitrate or chelating bidentate nitrate, respectively [20,21]. The intensity of the peaks increased with the time of exposure to NO_2_ gas and the intensities did not decrease even after the replacing NO_2_ flow with air flow.

In Figure 12, two strong peaks at around 1,535 cm^−1^ and a weak peak around 1,268 cm^−1^ were observed, which could be assigned to the chelating bidentate nitrate. From the spectra shown in Figures 11 and 12, we could suggest that exposure of NO_2_ gas to the Co_3_O_4_ surface formed the bridging bidentate, chelating bidentate, and monodentate nitrates at 100 °C and the chelating bidentate nitrate at 200 °C.

**Figure 13.**
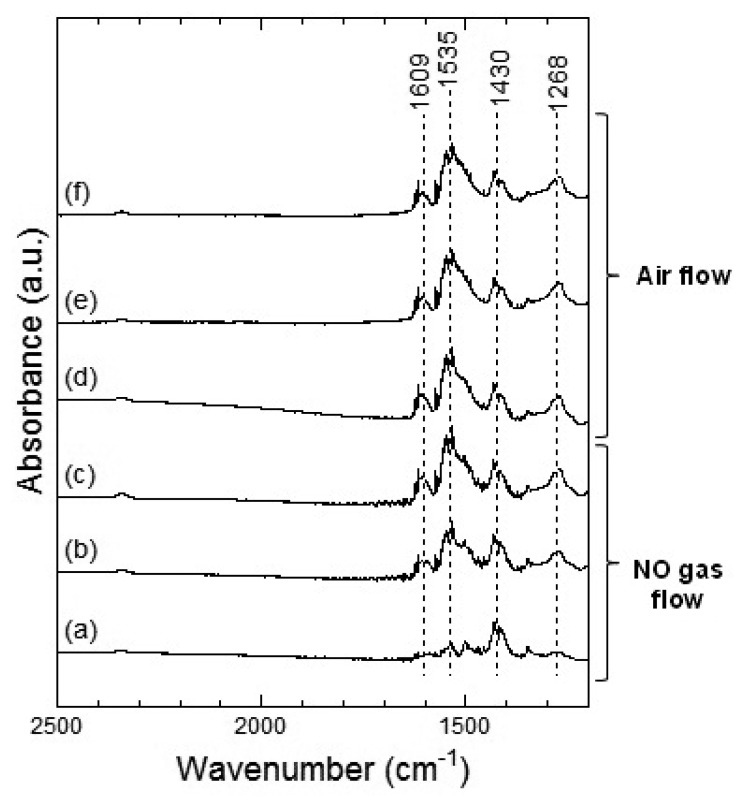
DRIFT spectra of Co_3_O_4_ powder at 100 °C. The powders were exposed to 1 ppm of NO in air for (**a**) 2 min, (**b**) 20 min, and (**c**) 50 min. After NO exposure, the powders were purged by air for (**d**) 1 min, (**e**) 30 min, and (**f**) 60 min.

**Figure 14.**
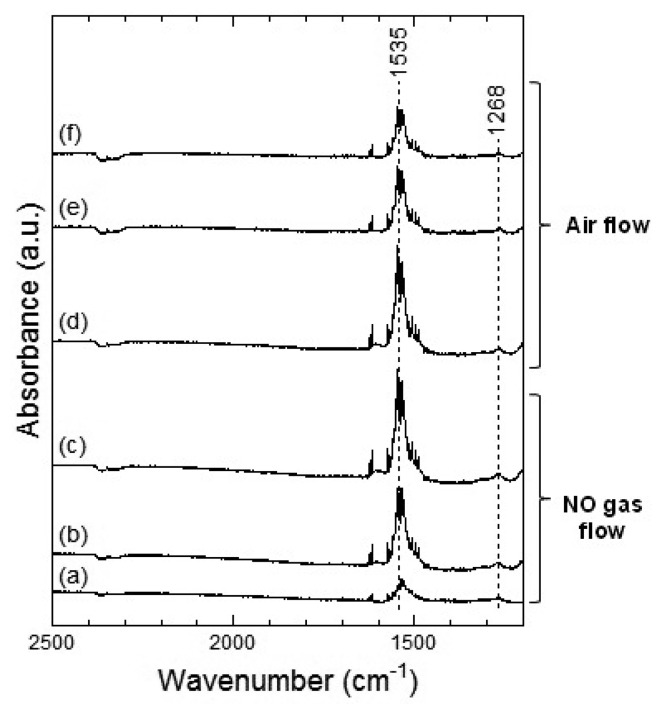
DRIFT spectra of Co_3_O_4_ powder at 200 °C. The powders were exposed to 1 ppm of NO in air for (**a**) 2 min, (**b**) 20 min, and (**c**) 50 min. After NO exposure, the powders were purged by air for (**d**) 1 min, (**e**) 30 min, and (**f**) 60 min.

Figures 13 and 14 show the DRIFT spectra of Co_3_O_4_ powder exposed to 1 ppm NO at 100 °C and 200 °C, respectively. The peak patterns shown in Figures 13 and 14, are similar to the patterns shown by the DRIFT spectra of Co_3_O_4_ powder exposed to NO_2_. Hence, the chemical states of the Co_3_O_4_ surface achieved on exposure to NO_2_ and NO seemed identical. Further, most peaks in the DRIFT spectra of Co_3_O_4_ exposed to air and on exposure to NO_2_ or NO gases were similar. Despite the changes observed in the resistance of Co_3_O_4_ on exposure to various atmospheres, there was no clear difference in the DRIFT spectra of Co_3_O_4_ powder exposed to various atmospheres. With the present data, it is difficult to discuss the origin of these contradictory results and we intend to investigate these in future.

As in the case of the DRIFT spectra of Co_3_O_4_ powder, no difference between NO_2_ and NO exposures could be observed in the case of the DRIFT spectra shown by WO_3_ powder. However, as shown in Figures 4 and 5, the sensor resistance of Co_3_O_4_ varied depended on the operating temperature (100 °C or 200 °C) and the gas species (NO_2_ or NO in air). It is to be noted that although the chemical state of the Co_3_O_4_ surface exposed to NO_2_ exposure was similar to that of the Co_3_O_4_ surface exposed to NO at 100 °C, the sensor resistance decreased on exposure to NO_2_ and increased on exposure to NO. With the change in the electron concentration on Co_3_O_4_ surface, the sensor resistance changed. The resistance of the sensor on exposure to NO_2_ decreased at 100 °C and increased at 200 °C. This can be viewed on the basis of the previous studies which report on the transitions from p-type to n-type behavior of several metal oxide sensors (or *vice versa*) [12,23–25]. In particular, Zhang *et al*. showed that the transition of WO_3_ was observed under 93 ppb NO_2_ exposure at working temperature above 130 °C [12]. A similar transition seemed to have occurred in the case of the Co_3_O_4_ sensor on exposure NO_2_ at 200 °C in this study. On the other hand, no transition of WO_3_ seemed to have occurred because NO_2_ concentration was higher than 0.5 ppm in this study. Although the transition would be due to the oxygen adsorption and formation of inversion layer at the metal oxide surface, further works are necessary to clear the transition mechanism. In this work, we could not examine the DRIFT spectra of WO_3_ or Co_3_O_4_ powder in the sample cell but the DRIFT spectra of WO_3_ or Co_3_O_4_ film on the Si substrate with a platinum comb-type electrode; the difference between the reactions of the powder and the film might be present. Due to the difference between the reactions of the powder and the film, some contradictory results in this work are supposed to be appeared. The chemical states of NO_2_ and NO on the sensor elements will be investigated in the future.

In this study, we have dealt with the sensor responses of WO_3_ and Co_3_O_4_ elements on exposure to NO_2_ or NO at different operating temperatures. In the case of the real-life application of the sensors, if the target gas consisting of an unknown mixture of NO_2_ and NO is to be analyzed by WO_3_ and Co_3_O_4_-based gas sensors, the results should be viewed carefully because the sensor response could be changed by the component ratio of NO_2_ and NO in NO*_x_*.

## 4. Conclusions

We have investigated the gas sensing properties of the sensor element that uses n-type WO_3_ or p-type Co_3_O_4_ toward NO_2_ and NO. Further, we also analyzed the chemical states of NO_2_ and NO on the semiconductor oxide surfaces. The resistance of the WO_3_ sensor at 100 °C and 200 °C increased with the concentration of NO_2_. The resistance of the WO_3_ sensor to NO exposure first increased and then immediately decreased within 10 min at 100 °C, while at 200 °C, the resistance of the sensor increased. Since the sensor resistance of Co_3_O_4_ exposed to NO increased at 100 °C and 200 °C, NO acted as a reducing reagent and oxidized to NO_2_. The sensor properties for NO exposure were consistent with the DRIFT spectra at 100 °C and 200 °C. On the other hand, since the sensor resistance of Co_3_O_4_ exposed to NO_2_ decreased at 100 °C, NO_2_ acted as an oxidizing agent and the sensor properties for NO_2_ exposure were consistent with the DRIFT spectra. At 200 °C, the sensor resistance of Co_3_O_4_ exposed to NO_2_ increased and the sensor properties for NO_2_ exposure were inconsistent with the DRIFT spectra. Interestingly, no clear differences between the chemical states of the metal oxide surface exposed to NO_2_ or NO could be detected from the DRIFT spectra of sensors based on either of the semiconductors. We think that NO was oxidized into NO_2_ and was adsorbed on the surface of WO_3_ or Co_3_O_4_ as NO_2_.
